# Children with Trans Parents: Parent–Child Relationship Quality and Psychological Well-being

**DOI:** 10.1080/15295192.2020.1792194

**Published:** 2020-08-04

**Authors:** Susan Imrie, Sophie Zadeh, Kevan Wylie, Susan Golombok

## Abstract

***Objective.*** Families with trans parents are an increasingly visible family form, yet little is known about parenting and child outcomes in these families. This exploratory study offers the first quantitative assessment of parent–child relationship quality and child socio-emotional and behavioral adjustment in families with a self-identified trans parent with school-aged children. ***Design.*** A sample of 35 families (37 trans parents, 13 partners, and 25 children aged 8–18 years) was recruited primarily through social media. Parents, children, and teachers were administered a range of standardized interview and questionnaire assessments of parent–child relationship quality, quality of parenting, psychological adjustment, and gender-related minority stress. ***Results.*** Parents and children had good quality relationships, as assessed by both parents and children, and children showed good psychological adjustment. Child age at the time the parent communicated their gender identity to the child was unrelated to child outcomes. ***Conclusions.*** Parents and children in trans parent families had good quality relationships and children showed good psychological adjustment. The findings of this exploratory study challenge commonly held concerns about the potentially negative effects on children of growing up with a trans parent.

## INTRODUCTION

The adult trans population in the United Kingdom is estimated at between 200,000 and 500,000 people (Government Equalities Office, 2018), with estimates in the United States of between 1 million and 1.4 million (Flores, Herman, Gates, & Brown, 2016; Meerwijk & Sevelius, 2017). Of the adult trans population, between 25% and 49% of individuals are believed to be parents (Dierckx, Motmans, Mortelmans, & T’sjoen, 2016), yet little is known about family functioning in trans parent families. With growing public and political awareness of equality issues affecting trans people (Stotzer, Herman, & Hasenbush, 2014; Women and Equalities Committee, 2016), families with trans parents are likely to become an increasingly visible family type.

In this article, the term trans is used to describe individuals whose gender identity is not the same as, or does not sit comfortably with, the sex they were assigned at birth. In keeping with its use by the largest UK-based nonprofit working solely with the trans community and those who impact on trans lives (Gendered Intelligence, 2019), this term is intended to be inclusive of (but not limited to) people who identify as trans, transgender, nonbinary, genderqueer, genderfluid, agender and gender nonconforming. Although the adequacy of such terminology has been challenged (Aguirre-Sánchez-Beato, 2018), all terms used in the study in general (and in this article in particular) have been subject to consultation with two nonprofit organizations with experience of working with this population in the UK, and therefore reflect the context in which this research was conducted.

Trans parents may form their families in a range of ways, including through biological parenthood, step-parenthood, adoption, fostering, and assisted reproduction, with an increasing number of options available to trans people wishing to become parents (Tornello, Riskind, Babić, & Tornello, 2019). Although research has begun to examine trans parents’ experiences of pursuing assisted reproductive treatment to start their families (e.g., James-Abra et al., 2015), older parents are more likely to have had children prior to gender transition.

The little empirical literature that exists on trans parenthood has focused primarily on families’ experiences of a parent’s transition, with a largely sociological approach (e.g., Dierckx, Mortelmans, Motmans, & T’Sjoen, 2017), or has been carried out by therapists (e.g., Veldorale-Griffin & Darling, 2016; White & Ettner, 2004). As such, the focus on trans parenthood has been rather narrow to date, with little examination of family functioning beyond a consideration of the effects of transition on the family system. Empirical evidence examining parent–child relationship quality and child psychological adjustment using standardized assessments is scarce and few studies have included school-aged children as informants. A review of trans parenting highlighted that the existing body of work is limited and would benefit from the inclusion of children’s perspectives (Hafford-Letchfield et al., 2019).

Despite this paucity of evidence on children’s outcomes and perspectives, assumptions about the presumed detrimental effects on children of growing up with a trans parent remain widespread. In the most extreme cases these assumptions have resulted in trans parents losing, or suffering restrictions of, their parental rights on the basis of their gender identity (Perez, 2009; Pyne, Bauer, & Bradley, 2015), under the belief that it was not in children’s best interests to have a relationship with their trans parent, or because the children would be ostracized from their community (J v B, 2017). A survey of over 6,000 trans people in the United States found that 29% of respondents with children reported an ex-partner limiting contact between the parent and child (Grant et al., 2011), and 10% of respondents in a Canadian survey of trans parents reported losing custody of their children (Pyne et al., 2015).

Concerns about the potential challenges faced by children with trans parents have tended to focus on the possible negative effects of these challenges on child psychological adjustment (Downing, 2013; Lev, 2010). For children who experience a parent’s transition, concerns have been raised about the child’s ability to negotiate a relationship with a parent with a different gender identity to that which they had originally known or assumed (Norwood, 2012). Furthermore, trans parent households may experience high levels of relationship and family conflict (Freedman, Tasker, & Di Ceglie, 2002; Haines, Ajayi, & Boyd, 2014), a factor known to negatively affect child adjustment through direct and indirect mechanisms, i.e., through the negative emotional and cognitive responses children may have when exposed to conflict, or by conflict in the parental relationship ‘spilling over’ into the parent–child relationship (Reynolds, Houlston, Coleman, & Harold, 2014). Children may also witness a parent’s distress, or the rejection of their parent by other family members, and some children have reported feeling caught in the middle of family relationships (Veldorale-Griffin, 2014). Thus, the maintenance of family relationships conducive to positive child development may be more challenging in families with trans parents.

Irrespective of whether they experience a parent’s transition or are born into families with trans parents, children may also experience stigma from peers, teachers, or from a wider society in which high levels of societal and systemic transphobia are present (Women and Equalities Committee, 2015; Dierckx et al., 2016). Furthermore, support for the children of trans parents has been described as practically non-existent (Stotzer et al., 2014), leaving children with little access to resources or information about their family type, and limited ability to contact other families in similar situations. As such, the child’s social environment may not provide adequate support to children in this family type (Haines et al., 2014; Veldorale-Griffin, 2014).

Existing research on child adjustment in families with trans parents has focused largely on children’s gender development (Chiland, Clouet, Golse, Guinot, & Wolf, 2013; Green, 1978, 1998) or the effects on children of a parent’s transition as reported by parents (Church, O’Shea, & Lucey, 2014; Veldorale-Griffin & Darling, 2016; White & Ettner, 2007), therapists (White & Ettner, 2004), or adults who have been reared by trans parents (Clarke & Demetriou, 2016; Veldorale-Griffin, 2014).

Freedman et al. (2002) examined family relationships and child psychological adjustment in a sample of 18 children aged 3–15 years who had been referred to a specialist gender identity disorder service, most of whom had been born before their parent transitioned. One child in the sample was reported as having depression, but 61% were found to have relationship difficulties with their parents (Freedman et al., 2002). In a follow-up study of 42 French children aged 1–12 years who had been born to trans men and their cisgender female partners following treatment with donor sperm, the children were described as “normal and happy,” although no details were provided of how the assessments were carried out (Chiland et al., 2013). A U.S. sample of 27 trans parents of 55 children aged 8–35 years reported that 35% of the children had a psychiatric disorder, rates that were similar to those found in the general population (White & Ettner, 2007). A recent international survey of trans and gender non-binary parents found parental division of labor to be unrelated to child behavioral outcomes (Tornello, 2020).

In terms of parent–child relationship quality, the children in Chiland et al.’s (2013) sample were described as securely attached, although no details of how attachment quality was assessed were provided. A survey of 48 U.S. trans parents and nine adult children found that two-thirds of parents and children reported either positive or no change in the parent–child relationship after beginning transition, and one-third of children and 15% of parents reported negative changes (Veldorale-Griffin, 2014). Similarly, a survey of 14 Irish trans parents (with 28 children aged between 5 and 34 years, 75% of whom were aware of the parent’s trans identity) found positive relationships with 25 of the children, as reported by parents in interviews and assessed using a questionnaire measure of relationship problems (Church et al., 2014).

In families in which children experience a parent’s transition, several protective and risk factors have been identified. First, the quality of the relationship between parents has been identified as an important influence on child well-being, with transphobic attitudes of the second parent linked to negative outcomes for children (Freedman et al., 2002; Hines, 2006; White & Ettner, 2004), and lower levels of post-transition conflict between parents predicting positive relationships between trans parents and children (White & Ettner, 2007). Second, experience of social stigma has been identified as a risk factor, with adult children reporting fear of stigmatization and bullying as one of the most common stressors associated with their parent’s transition (Veldorale-Griffin, 2014). It has also been suggested that the child’s age at the time of transition may be a risk factor, with older children believed to be less accepting than younger children (Veldorale-Griffin, 2014; White & Ettner, 2007), although this conclusion is drawn only from parent and therapist reports. Open communication between parents and children, continuity in parental behavior and family structure, acceptance by the other parent and the child’s peers, and the meaning attributed to the transition have been identified as factors that can aid adaptive family functioning during a parent’s transition (Dierckx et al., 2017).

### Minority Stress Theory and Children with Trans Parents

It has been well established in the minority stress literature that chronic psychological stress resulting from membership of a stigmatized social group is linked to poorer psychological health outcomes in sexual minority persons (Meyer, 2003; Rostosky & Riggle, 2017). Meyer (2003) highlighted multiple ways in which minority stress might be experienced, including via distal (external) stressors (e.g., experience of rejection, violence or discrimination) and via proximal (internal) stressors (including fear of discrimination, the internalization of negative beliefs about one’s identity, and the stress of having to conceal that identity). Resilience factors that can alleviate the effects of the experience of minority stress on mental health include experiencing emotional and social support from others who share the minority identity, community membership, and pride in one’s minority identity (Meyer, 2003). Other work has expanded the minority stress model for trans and gender nonconforming people. The gender minority stress framework (Testa, Habarth, Peta, Balsam, & Bockting, 2015) has been used to demonstrate similar relations between minority stressors and resilience factors and mental health outcomes in trans samples (Bockting, Miner, Swinburne Romine, Hamilton, & Coleman, 2013; McLemore, 2018; Testa et al., 2012).

Given that trans individuals experience high rates of minority stressors irrespective of their country of residence (Grant et al., 2011; Testa et al., 2012; Women and Equalities Committee, 2016), it is not inconceivable that the children of trans parents could also be considered members of a minority group and may also experience minority stress. Connolly (2006) argued that stigma surrounding trans identities may influence trans people’s immediate and extended families in covert and overt ways, for example, family members may internalize transphobic beliefs and this may influence their interactions with their trans family member. Several studies have found that children with trans parents have experienced challenges related to societal transphobia, including harassment by peers (Dierckx et al., 2017; Freedman et al., 2002; Haines et al., 2014). Others have reported children’s fear of stigmatization and bullying (Veldorale-Griffin, 2014) and that children may attempt to conceal their trans parent’s identity to some extent (Church et al., 2014; Haines et al., 2014). Whereas all of these experiences could potentially directly affect children’s psychological well-being as conceptualized by the minority stress model, it is also possible that dyadic minority stress processes may be relevant in this sample. Stress contagion, a type of stress proliferation, occurs when an individual’s experience of minority stress at an individual level negatively affects a partner’s psychological health (Pearlin, 1999) and has been demonstrated in both same-sex couples (Frost et al., 2017) and among trans women and their cisgender male partners (Gamarel, Resiner, Laurenceau, Nemoto, & Operario, 2014). It is plausible that minority stress experienced by a parent could also affect child adjustment, directly through stress proliferation and indirectly via parenting. Experiences of parent-experienced minority stress relate to adjustment problems in children, specifically higher parent-reported stigmatization is associated with hyperactivity in boys and low self-esteem in girls aged 4–8 years in lesbian mother families (Bos & van Balen, 2008) and with higher rates of externalizing problems in 4-to 8-year-olds in lesbian mother and gay father families (Golombok et al., 2018). Similarly, stigmatization reported by 17-year-olds in lesbian mother families was associated with higher rates of both externalizing and internalizing problems, but close positive parent-adolescent relationships buffered the effects of stigmatization on adolescent adjustment (Bos & Gartrell, 2010). To date, no empirical research has examined whether trans parents’ experiences of minority stress are related to child adjustment or parent–child relationship quality.

### The Present Study

The current study aimed to provide the first exploratory, quantitative assessment of parent–child relationship quality and child socio-emotional and behavioral adjustment in families with a self-identified trans parent. An exploratory design is deemed appropriate when addressing newly emerging social issues for which a slim evidence base exists (Henry, 2013). The study also aimed to investigate the factors associated with parent–child relationship quality and child adjustment in this family type, including whether the child’s age when the parent communicated their gender identity to them was related to child outcomes.

We chose to examine the child’s age at parental communication of gender identity rather than child’s age at parental transition (as has been used in other studies) as it most accurately reflected the experiences of the participants. Some parents did not consider themselves as having transitioned but as having communicated their gender identity to their child (which was different to the gender identity their child had assumed them to hold). Other parents considered themselves as having transitioned or having started a process of transition and had not communicated their gender identity to their child. Due to the heterogeneity of parents’ experiences, the most relevant variable for understanding children’s outcomes was thus considered to be the child’s age at parental communication of gender identity.

## METHOD

### Participants

Thirty-five families with at least one self-identified trans parent participated in the study, including 37 trans parents, 13 cisgender parents, and 25 children (aged 8–18 years). Information on the number of family members in each household who participated is provided in Table 1. The data form part of a larger study that included interviews with children aged 4–18, reported elsewhere (Zadeh, Imrie, & Golombok, 2019).

**Table 1.** Participant family structure, family formation method, and country of residence

	No. of Families (*N* = 35)
Family members participating	
1 trans parent	14
1 trans parent, 1 child	5
1 trans parent, 1 cis parent, 1 child	5
1 trans parent, 1 cis parent	4
1 trans parent, 1 cis parent, 2 children	3
2 trans parents, 2 children	2
1 trans parent, 2 children	1
1 trans parent, 1 cis parent, 3 children	1
Parenting arrangement for target child	
Child living with both legal parents	16
Shared parenting: shared equally between two households	4
Shared parenting: child mainly with trans parent	6
Shared parenting: child mainly with other parent	8
Child living with partner	1
Family formation	
Unassisted conception	26
Assisted reproductive technologies	5
Adoption	3
Long-term foster care	1
Country of residence	
England	26
Scotland	3
Wales	3
United States	2
Ireland	1

Families were recruited through social media. Two nonprofit organizations (Stonewall and Gendered Intelligence) posted an advert on their Twitter and Facebook pages and disseminated the advert through their links with other organizations working with trans adults. Online recruitment was also carried out through Scottish Trans Alliance and GIRES. As the study was exploratory, the inclusion criteria were broad; the advert asked ‘trans parents with a child aged 0–18 years’ interested in a study of family life to contact the researchers to receive further information. Fifty-four people contacted the researchers, of whom four were ineligible, and 33 participated in the study (participation rate = 66%). Two additional families were recruited through an event for LGBTQ+ families in the United States.

Data collection with parents focused on a target child aged 4–18 years (*M* = 11.57, *SD* = 4.10). In 28 families, the target child had been born before the trans parent communicated their gender identity to others, and in seven families the child had been born afterward. Trans parents had communicated their gender identity to the target child between 3 months and 10 years prior to data collection (*M* = 3.79 years, *SD *= 3.02), and the child had been aged between 2 and 16 years old at the time (*M* = 9.14 years, *SD* = 4.12). Six trans parents had not yet communicated their gender identity to their children. In four families, trans parents had a second child with whom they had no contact.

Parents who had responded to the advert chose whether to invite their child(ren) and partner (where applicable) to participate in the study. In 23 families, parents lived with a partner, of whom 15 (65%) provided interview and/or questionnaire data. In the 35 families, trans parents had 46 children who were eligible to participate (aged between 8 and 18 years, in contact with the parent). In two families, parents had not yet communicated their gender identity to their children so three eligible children were not invited to participate. Three further children were not present when their parents arranged the data collection visit, and one child’s other parent did not give consent for her to participate. The remaining 10 children declined to participate. Twenty-five children from 19 families provided questionnaire data (*M* = 12.64, *SD *= 2.89).

Trans parents were aged 33–59 years (*M* = 43.97, *SD* = 6.06) and cis parents were aged 29–54 (*M* = 42.08, *SD* = 5.95). Parents were asked to describe the parenting arrangements for their child(ren) and their method of family formation (see Table 1) and to describe their gender identity in their own words (Table 2). Most trans parents (77%, *n* = 27) identified their ethnicity as White British, and half (50%, *n* = 16) had a higher educational qualification (Table 2).

**Table 2.** Participant self-identified gender identity and demographic information

	Trans Parents (*n* = 37)	Cis Parents (*n* = 13)	Children (*n* = 25)
Gender identity			
Trans woman	13	0	0
Female	6	8	9
Trans man	4	0	0
Nonbinary/nonbinary trans	3	0	1
Female/trans female	2	0	0
Genderfluid	1	0	1
Genderqueer	1	0	1
Agender	1	0	0
Transsexual	1	0	0
Trans woman/nonbinary	1	0	0
Male to female	1	0	0
Trans/gender nonconforming	1	0	0
Woman with a trans history	1	0	0
Male	1	3	13
Cisgender female	0	2	0
Ethnicity			
White British	27	12	
White Irish	2	0	
Other White background	6	1	
Education			
Higher education	16	11	
School education	16	2	

### Procedures

All families were visited at home (or at a location of their choosing) by one of the two researchers. Researchers had received additional training from Gendered Intelligence, a charity working with the trans community. Two parents and one child were interviewed by phone due to geographic location. Parents and children were administered audio-recorded standardized interviews and batteries of standardized questionnaires. Each home visit lasted between 1.5 and 3 hr. Ethical approval was granted by the University of Cambridge Psychology Research Ethics Committee. Data were collected between February 2017 and December 2018.

## MEASURES

### Parenting Quality

Parents were administered an adaptation of a semi-structured interview designed to assess quality of parenting (Quinton & Rutter, 1988). Parents are asked to provide detailed accounts about the child’s behavior and the parent’s response to it, with variables rated using a standardized coding scheme based on a detailed coding manual. The following variables were coded: (1) *expressed warmth* from 1 (*none*) to 6 (*high*) took into account the parent’s facial expressions, gestures, tone of voice and interest when talking about the child; (2) *quality of interaction* from 1 (*very poor*) to 5 (*very good)* was based on the extent to which the parent and child wanted to be together, enjoyed each other’s company and showed affection to each other; (3) *sensitive responding* from 1 (*none*) to 5 (*very sensitive*) assessed the parent’s ability to recognize and respond appropriately to the child’s needs; (4) *level of battle* examined the level of parent–child conflict from 1 (*no confrontations*) to 4 (*major battle)*; (5) *frequency of battle* assessed the frequency of parent-child conflict from 1 (*never*) to 6 (*a few times a day*); and (6) *criticism* assessed the degree of the parent’s criticism of the child from 1 (*no criticism*) to 5 (*considerable)*. One-third of interviews were rated by a second coder. Intraclass correlations for the variables were as follows: expressed warmth (.76), quality of interaction (.70), sensitive responding (.66), level of battle (.67), frequency of battle (.71).

### Parent-Child Relationship Quality

Parents and children were administered the 24-item version of the Parental Acceptance Rejection Questionnaire, which assesses parental acceptance/rejection in the parent–child relationship (PARQ; Rohner, 2005). The PARQ comprises four subscales (warmth-affection, hostility-aggression, rejection, neglect-indifference), scores from which are summed to provide a total score. Scores of 24 indicate highest acceptance and lowest rejection, with scores of 96 indicating highest rejection and lowest acceptance. Scores above 60 (the scale mid-point) indicate higher levels of rejection than acceptance. Data on parent acceptance/rejection were obtained from both parents and children; parents completed the PARQ regarding their feelings toward the child, and the child completed the PARQ regarding their perception of each of their parent’s feelings toward them. The PARQ has good internal consistency, and convergent and discriminant validity (Rohner, 2005). Cronbach’s alpha for parent-reported scores was 81. Cronbach’s alpha for child-reported scores for their relationship with their trans parent was .88 and for their relationship with their cis parent was .86.

### Child Psychological Adjustment

#### Strengths and Difficulties Questionnaire

Child psychological adjustment was assessed using the Strengths and Difficulties Questionnaire (SDQ; Goodman, 1997) administered to parents. The SDQ is a behavioral screening questionnaire that yields a “total difficulties” score from 0 to 40, with higher scores indicating greater adjustment problems. Scores of 13 and below are classified as within the normal range, scores of 14–16 classified as borderline, scores of 17–19 classified as high, and scores of 20–40 classified as very high. Where parental consent was obtained, an independent assessment of the target child’s psychological adjustment was obtained from an SDQ administered to the child’s teacher. Teachers were not informed of the family type being studied, as some parents had not communicated their gender identity to the child’s school. Teachers were told that the child was taking part in a study of child development and family relationships and that their responses would not be reported back to the family. Questionnaires were sent to 17 teachers and 11 were returned (response rate = 65%). The SDQ has good internal consistency, test-retest and inter-rater reliability, and discriminates well between psychiatric and non-psychiatric samples (Goodman, 1997). A review of the reliability and validity of the SDQ based upon scores of over 130,000 children from 48 studies found the SDQ to have strong psychometric properties (Stone, Otten, Engels, Vermulst, & Janssens, 2010). Cronbach’s alpha for the present parent sample was .73.

#### Rosenberg Self Esteem Scale

Children aged 12–18 were administered the Rosenberg Self-Esteem Scale to provide a measure of self-esteem (RES; Rosenberg, 1965). The questionnaire has 10 items and scores range from 10 to 30, with higher scores indicating higher self-esteem. Scores between 15–25 are considered within the normal range, and scores below 15 indicate low self-esteem. The scale has high internal consistency (Schmitt & Allik, 2005). Cronbach’s alpha for the present sample was .92.

#### Ratings of Psychiatric Disorder

The presence of child psychiatric disorder was assessed during the interview with the parent using a standardized procedure (Rutter, Cox, Tupling, Berger, & Yule, 1975). Parents were asked to provide detailed descriptions of any behavioral or emotional problems shown by the child. Descriptions included information about the severity, frequency, precipitants, and course of particular behaviors over the last year, and these interview extracts were transcribed and rated by a child psychiatrist unaware of family type. Ratings made blind by a child psychiatrist and those made by social scientists have a high level of reliability (*r* = .85), and validity has been established through a high level of agreement between mothers’ assessments of their child’s behavioral or emotional difficulties and interview ratings of child psychological problems (Rutter et al., 1975). Type of disorder was identified as: conduct disorder, emotional disorder, mixed disorder, developmental disorder, ADHD, psychotic disorder, or other disorder. Ratings were made on a 4-point scale: 0 (*no disorder)*, 1 (*dubious or trivial disorder*), 2 (*slight disorder*), 3 (*definite*/*marked disorder)*.

### Parent Psychological Well-being

#### Beck Depression Inventory-II

Parents were administered the Beck Depression Inventory-II (BDI-II; Beck, Steer, & Brown, 1996) to assess the presence of depression. The 21-item questionnaire yields scores between 0–63, with scores of 13 and below indicating minimal depression, 14–19 indicating mild depression, 20–28 indicating moderate depression and 29–63 indicating severe depression. The BDI-II has excellent reliability and validity (Dozois, Dobson, & Ahnberg, 1998). Cronbach’s alpha for the present sample was .93

#### Parenting Stress Index

Parents were administered the short form of the Parenting Stress Index (PSI-SF; Abidin, 1995), to assess stress associated with parenting. The 36-item questionnaire yields total scores from 36–180, with higher scores reflecting greater parenting stress. A total score above 90 indicates that a parent is experiencing clinically significant levels of stress (Abidin, 1995). Test-retest reliability for the questionnaire is high. Concurrent and predictive validity have been demonstrated for the full-length questionnaire, and the short form correlates very highly with the full-length version (Abidin, 1995). Cronbach’s alpha for the present sample was .91.

#### Multidimensional Scale of Perceived Social Support

The Multidimensional Scale of Perceived Social Support was administered to parents to assess their perceived levels of social support (MSPSS; Zimet, Dahlem, Zimet, & Farley, 1988). The 12-item questionnaire comprises three subscales that measure the perceived adequacy of support from three sources: family, friends, and significant other. Each item is rated on a 7-point scale with higher scores indicating higher perceived social support. Zimet et al. (1988) suggested that mean scale scores between 1–2.9 can be classified as low support, 3–5 as moderate support and 5.1–7 as high support. The MSPSS has good validity (Dahlem, Zimet, & Walker, 1991) and good test-retest reliability. Cronbach’s alpha for the present sample was .92.

#### Gender Minority Stress and Resilience Measure

Several subscales from the Gender Minority Stress and Resilience Measure were administered to trans parents to measure aspects of gender minority stress and resilience (GMSR; Testa et al., 2015). The subscales assessed gender-related discrimination, gender-related rejection, non-affirmation of gender identity, pride and community connectedness. Range of scores for the subscales are as follows, with higher scores indicating higher presence of the construct of interest: Gender-related discrimination (0–5), gender-related rejection (0–6), non-affirmation of gender identity (0–24), pride (0–32), community connectedness (0–20). The measure has good reliability and validity for use with trans and gender nonconforming populations (Testa et al., 2015). Cronbach’s alpha for the present sample was .81.

Descriptive statistics for measures of parenting quality, parent–child relationship quality, and psychological well-being are shown in Table 3.

**Table 3.** Descriptive statistics for measures of quality of parenting, parent–child relationship quality, and psychological well-being

	Trans Parents (*n* = 34)		Cis Parents (*n* = 12)		Children (*n* = 25)		Teachers (*n* = 11)	
	*M*	*SD*	*M*	*SD*	*M*	*SD*	*M*	*SD*
Beck Depression Inventory-II	12.74	11.02	7.92	6.23				
Parenting Stress Index (short-form)	69.91	18.65	69.58	14.54				
Multidimensional Scale of Perceived Social Support	5.24	1.39	5.74	1.23				
Gender Minority Stress and Resilience Measure								
Discrimination	1.84	1.27						
Rejection	2.44	1.44						
Non-affirmation	11.25	7.20						
Pride	19.16	8.08						
Community connectedness	13.56	4.23						
	Trans Parents (*n* = 35)		Cis Parents (*n* = 11)					
	*M*	*SD*	*M*	*SD*				
Strengths and Difficulties Questionnaire	9.03	4.57					6.09	4.89
Positive parenting	.08	.97	−.15	.93				
Negative parenting	−.06	.94	.28	.52				
Parental Acceptance-Rejection Questionnaire								
*Parent rating for childª*	30.75	5.75	31.13	6.88				
*Child rating for trans parent*					32.60	6.92		
*Child rating for other parent^b^*					30.71	5.99		
Rosenberg Self-Esteem Scale^c^					19.06	6.30		

ª Trans parents *n* = 28, cis parents *n* = 8

*^b^n* = 21

*^c^n* = 18

### Support From Child’s Other Parent

The interview with the trans parent included a semi-structured section, which examined the parent’s experience of communicating their gender identity to their family and was informed by the existing literature and the exploratory nature of the study. Parents were asked about how they had communicated their gender identity to their child(ren) and partner (where applicable), their family’s responses, and the effect of this experience on family relationships. This section of the interview was transcribed and transcripts were analyzed using a text-driven qualitative content analysis approach (Krippendorf, 2013). Two codes were generated from an initial reading of the data. The interviews were then rated according to these codes, and frequency counts calculated. The following ratings were made regarding the trans parent’s support from the child’s other parent: (1) level of conflict with child’s other parent and (2) level of support from child’s other parent. Level of conflict was rated on a 4-point scale from 1 (*amicable relationship*) to 4 (*high conflict/relationship breakdown*). Support from the other parent was coded on a 3-point scale from 1 (*not supportive)* to 3 (*fully supportive)*. One-third of transcripts were rated by a second coder. Intraclass correlations were as follows: level of conflict (0.97), level of support (1).

### Analytic Plan

First, exploratory factor analysis (using principal axis factoring with direct oblimin rotation) was conducted with the interview variables related to parenting quality to establish whether they reflected an underlying construct of parenting quality. Two factors, both with item loadings of at least 0.6, explained 71% of the total variance. The first factor (comprising expressed warmth, quality of interaction, and sensitive responding) was labeled *positive parenting*, and the second factor (comprising criticism, level of battle, and frequency of battle) was labeled *negative parenting*. The correlation between the two factors was *r*(46) = −.40, *p* < .001, showing a moderate negative relation between them.

Parents’ and children’s scores on the questionnaire assessments of psychological adjustment and parent–child relationship quality were compared to clinical cutoff scores. Where comparisons with population norms were possible, one-sample *t-*tests were used. Correlations were used to explore relations between parental psychological well-being scores and scores on the gender minority stress and resilience measure. To explore relations between child adjustment, parent-child relationship quality, child age when the parent communicated their gender identity to them, and parent-experienced gender minority stress, correlations between these variables were examined.

Linear regressions were carried out to examine factors predicting child adjustment and parent–child relationship quality. Establishing which family process variables to include in the model was guided firstly by the existing literature. Second, family process variables that were theoretically associated with the outcome variable were checked to see whether they correlated with the outcome variable. Variables that were significantly associated with the dependent variable were included in the multiple regression analyses using a forced entry method.

Where parental psychological health data are presented, all trans parents’ scores are included. Findings on target child psychological adjustment, trans parent–child relationship quality, and regression analyses use data from one parent per family only. In households with one trans parent or one trans parent and one cis parent, the trans parent’s ratings were used. In the two households with two trans parents, the ratings made by the child’s biological parent were used.

## RESULTS

### Parent-Child Relationship Quality

Parent-reported scores on the Parental Acceptance-Rejection Questionnaire were all below the mid-point of the scale, showing that parents rated the parent–child relationship as more accepting than rejecting. Trans parents’ mean score (*M* = 30.75, *SD *= 5.75) was well below the scale mid-point, indicating good parent–child relationship quality. Cis parents also rated their relationships with the children as more accepting than rejecting, with all scores below the mid-point of the scale and mean scores indicating good relationship quality (*M* = 31.13, *SD* = 6.88). All children rated their trans parent and their other parent as more accepting than rejecting, with all scores for both parents below the scale mid-point. There was no difference between the way in which children viewed the quality of their relationship with their trans parent (*M* = 32.60, SD = 6.92) and their other parent (*M* = 30.71, *SD* = 5.99), as assessed using a paired-sample *t*-test, *t*(20) = 1.31, *p* = .21, *d* = .29.

### Parent Psychological Well-being

Parents’ scores on the BDI-II showed that 21 (60%) trans parents and 10 (83%) cis parents scored within the normal range. Five (14%) trans parents and one (8%) cis parent had scores indicating mild depression, five (14%) trans parents’ and one (8%) cis parent’s scores indicated moderate depression, and four (11%) trans parents had scores indicating severe depression. Thirty-one (89%) trans parents and ten (83%) cis parents scored within the normal range on the PSI-SF. Four (11%) trans parents and two (17%) cis parents had scores indicating clinically significant levels of stress. Twenty-one (60%) trans parents and nine (75%) cis parents had high perceived social support, eleven (31%) trans parents and three (25%) cis parents had moderate perceived social support, and three (9%) trans parents had low perceived social support. For trans parents, non-affirmation of gender identity was associated with parenting stress, *r*(31) = .41, *p* = .02, and perceived social support, *r*(31) = −.43, *p* = .01 (Table 4). Parents who experienced greater levels of non-affirmation experienced higher parenting stress and reported lower levels of perceived social support. As non-affirmation was also associated with the time since the parent had communicated their gender identity to the child, a partial correlation was carried out to control for time since communication. When time since communication was controlled for, the associations between both non-affirmation of gender identity and parenting stress, *r*(23) = .30, *p* = .14, and social support, *r*(23) = −.34, *p* = .10, were non-significant.

**Table 4.** Sample correlations

	1.	2.	3.	4.	5.	6.	7.	8.	9.	10.	11.	12.	13.
1. Strengths and Difficulties Questionnaire	-												
2. Parental Acceptance-Rejection Questionnaire	.21	-											
3. Parenting Stress Index	.44*	.40*	-										
4. Beck Depression Inventory-ii	.45**	.17	.52**	-									
5. Multidimensional Scale of Perceived Social Support	−.36*	−.03	−.41*	−.58***	-								
6. Positive parenting	−.01	−.53**	−.26	−.34*	−.03	-							
7. Negative parenting	.10	.57**	.52**	.33	−.03	−.53**	-						
8. Gender Minority Stress and Resilience Scale: discrimination	−.04	.07	.23	.29	−.03	−.18	.18	-					
9. Gender Minority Stress and Resilience Scale: rejection	.09	−.30	.05	.22	−.05	.02	−.05	.52**	-				
10. Gender Minority Stress and Resilience Scale: non-affirmation	.18	.13	.41*	.20	−.43*	.04	.02	.08	−.02	-			
11. Gender Minority Stress and Resilience Measure: pride	−.12	−.29	−.16	.35	.06	.22	.01	−.03	.28	−.05	-		
12. Gender Minority Stress and Resilience Scale: community connectedness	.02	−.21	−.14	.05	−.23	.04	−.04	.06	.19	−.12	.28	-	
13. Time since parental communication of gender identity to child	−.05	−.19	−.28	.01	.31	.19	−.17	.33	.05	−.48*	−.31	−.23	-
14. Child age at parental communication of gender identity	−.13	.08	.12	−.02	−.09	−.22	−.01	−.42*	−.03	.26	−.06	.03	−.62**

* *p* <.05. ** *p* <.01. *** *p* <.001.

### Child Psychological Adjustment

Parent-reported scores on the SDQ showed that the majority of children (86%, *n* = 30) scored within the normal range for total difficulties, indicating an absence of emotional and behavioral problems. When total difficulties scores were compared to normative SDQ data, the current sample did not differ from norms, *t*(34) = .81, *p* = .42, *d* = .14, suggesting that the children’s scores were in line with scores expected in a typical sample of school-aged children. Similarly, teacher-rated SDQ scores did not differ from norms, *t*(10) = −.35, *p* = .74, *d* = .02, showing that teachers also rated children as showing good psychological adjustment.

Child-reported scores on the Rosenberg Self-Esteem Scale showed that most (83%, *n* = 15) children scored within the ranges indicating average or above-average self-esteem. Twelve children (66%) scored in the normal range, three (17%) had scores indicating higher than average self-esteem, and three (17%) had scores indicating lower than average self-esteem.

With respect to the ratings by the child psychiatrist, four children showed a definite/marked disorder (two with emotional disorders; one with mixed developmental, conduct and emotional disorder; and one with mixed hyperkinetic, emotional, conduct, and developmental disorder).

### Support From Child’s Other Parent

Trans parents’ reports of their relationship with the child’s other legal parent indicated that in 24 families (69%) the relationship between the child’s parents was categorized as amicable. In four (11%) families, parents experienced some conflict, in two (7%) families parents had moderate levels of conflict, and in five (14%) families there were high levels of conflict or relationship breakdown between the child’s parents. Of the 31 families where the child’s other parent knew about the trans parent’s gender identity, 22 (71%) were fully supportive, 7 (23%) were somewhat supportive, and 2 (6%) were not supportive.

### Relation between Parent-Child Relationship Quality and Timing of Parent Communication of Gender Identity to Child

In the families in which parents had come out as trans after having children, and had also communicated their gender identity to their child, correlations between parent and child PARQ scores and the timing of communication variables (child’s age when parent communicated their gender identity to them, amount of time passed since) were examined to explore the relations between parent–child relationship quality and the timing of the parent’s communication about their gender identity. Child’s age at parent’s communication was not significantly associated with either parent-reported, *r*(23) = .08, *p* = .72, or child-reported, *r*(23) = .30, *p* = .14, parent–child relationship quality. In addition, the amount of time that had passed since the parent communicated their gender identity to the child was not associated with either parent-reported, *r*(23) = −.19, *p* = .35, or child-reported, *r*(23) = −.27, *p* = .20, relationship quality.

### Relation between Child Adjustment and Timing of Parent Communicating Their Gender Identity to Child

In the families in which parents had come out as trans after having children, and had also communicated their gender identity to their child, correlations between child SDQ scores and the timing of communication variables child’s age when parent communicated their gender identity to them, amount of time passed since were examined to explore the relation between child adjustment and the timing of the parent’s communication about their gender identity. The age of the child at the time of communication, *r*(25) = −.13, *p* = .51, and the number of years that had passed since, *r*(25) = −.05, *p* = .80, were not associated with SDQ total difficulties scores (see Table 4).

### Relation between Child Adjustment and Parent-Experienced Gender Minority Stress and Resilience

Correlations between SDQ scores and trans parents’ scores on the GMSR were examined to explore whether child adjustment was related to parents’ experiences of gender minority-related stress or resilience factors. As can be seen in Table 4, no significant correlations were found between child adjustment and any of the GMSR subscales.

### Predictors of Parent-Child Relationship Quality

Correlations were examined between PARQ total scores and the parental psychological health, quality of parenting, parental conflict, and gender minority stress variables to identify which variables to include in the regression predicting parent–child relationship quality. Correlations can be found in Table 4. Parenting stress, positive parenting, and negative parenting were all correlated with PARQ scores. As the positive and negative parenting variables were moderately correlated only positive parenting was included in the regression. A hierarchical multiple regression analysis was carried out. Parenting stress was added in the first block, followed by positive parenting in the second block. The overall model was significant, *F*(2, 25) = 7.50, *p* = .003, explaining 37.5% of the variance (adjusted *R*^2^ = 32.5% of variance) in parent–child relationship quality. Parenting stress and positive parenting each contributed significantly to the model and were similar in their prediction strength (see Table 5) such that higher levels of parenting stress and lower levels of positive parenting each predicted poorer relationship quality.

**Table 5.** Hierarchical multiple regression analysis predicting parent–child relationship quality

Step	Variable	*B*	*SE B*	*ß*
1.	Parenting stress	.120	.053	.403
2.	Parenting stress	.093	.048	.311
	Positive parenting	−2.728	.936	−.470*

*Note. R2 *=.133 for Step 1: *ΔR2 *=.212 for Step 2 (*p* =.01)

* *p* <.05

### Predictors of Child Adjustment

Correlations were examined between the SDQ total difficulties scores and the parental psychological health BDI-II, PSI, MSPSS, parent–child relationship quality (PARQ), quality of parenting positive parenting, negative parenting, parental conflict, and gender minority stress variables to identify which variables to include in the regression analyses. Correlations are shown in Table 4.

The parent psychological well-being variables of depression, parenting stress, and perceived social support were all correlated with SDQ total difficulties scores (and all correlated with each other). A linear regression analysis was run to establish the common and unique contributions of each parental psychological well-being dimension to the variance in child adjustment, with parental depression, parenting stress, and perceived social support entered into the same block. The results of the analysis predicting SDQ scores are shown in Table 6. The model predicted 26.5% of the variance in SDQ total difficulties scores, *F*(3, 29) = 3.49, *p* = .03 (or adjusted *R*^2^ = 18.9% of variance), although no variable contributed uniquely to the model. Higher parental depression, higher parenting stress, and lower perceived social support together predicted higher levels of child adjustment problems.

**Table 6.** Multiple regression analysis predicting child adjustment (SDQ total difficulties)

Step	Variable	*B*	*SE B*	*ß*
1.	Parenting stress	.066	.047	.262
	Depression	.104	.087	.250
	Perceived social support	−.361	.654	−.109

*Note. R2 *=.265

## DISCUSSION

This exploratory study aimed to provide a quantitative assessment of parent–child relationship quality and child adjustment in families with trans parents, an understudied family type. Overall, the study found good quality relationships between parents and children and low levels of behavioral and emotional problems in children with trans parents.

The finding that parent–child relationships were of good quality in trans parent families is in line with the small literature in this area that found positive parent–child relationships between parents and school-aged and adult children (Chiland et al., 2013; Church et al., 2014; Veldorale-Griffin, 2014). That high-quality relationships were reported from both parents’ and children’s perspectives gives greater weight to the finding. In-depth interviews with children from the same sample similarly highlight that having a trans parent has little or no impact on how children feel about the parent–child relationship (Zadeh et al., 2019). The current study is the first to include school-aged children as informants on the parent–child relationship in trans parent families, in a field that has until recently only included adult reports. Parental acceptance as rated on the Parental Acceptance and Rejection Questionnaire is known to promote a range of positive outcomes cross-culturally (Putnick et al., 2015), including psychological adjustment (Rohner, 2010) and child prosocial behavior (Putnick et al., 2018), and as such the current findings indicate the likelihood of good future outcomes for the children in the current sample.

Contrary to commonly held concerns about the potentially detrimental effects for children of growing up with a trans parent, the children and adolescents in the current sample showed good psychological adjustment and did not show elevated rates of problems in comparison to population norms. Good psychological adjustment was found across several measures and as assessed by multiple informants (parents, children, and teachers). The ratings of disorders by the child psychiatrist corroborated these findings. The 11% of children who showed a definite/marked disorder is lower than the U.K. population norm of 12.8% (Sadler et al., 2018). As the child psychiatrist was unaware of the family type being studied, these findings provide important validation for the parents’ reports. The findings are in line with the small body of existing literature that similarly found good psychological adjustment in children with trans parents (Chiland et al., 2013; Freedman et al., 2002) and strengthen the empirical basis by offering an assessment using one of the most widely used screening instruments of psychiatric disorders (Vugteveen, De Bildt, Hartman, & Timmerman, 2018).

That children’s SDQ scores were unrelated to the age at which their parent had communicated their gender identity to them is of particular interest, given that previous literature suggested that older children may find it harder to adjust to a parent’s transition (Veldorale-Griffin, 2014; White & Ettner, 2007). In the current sample, child’s age at time of the parent’s communication was unrelated to child psychological adjustment. It is possible that this difference in findings is due to a difference in methodologies, in that the current study used a validated, quantitative assessment of child adjustment. It is also possible that the lack of adjustment problems shown by children, and the lack of relation between child’s age at parent’s communication of gender identity and child adjustment, reflects a broader trend among adolescents of a growing awareness and acceptance of gender diversity (Bragg, Renold, Ringrose, & Jackson, 2018). As SDQ scores were also unrelated to the amount of time that had passed since the parent communicated their gender identity to the child, it was also not the case that children who had found out more recently about their parent’s gender identity showed higher rates of problems.

Contrary to predictions made in line with minority stress theory and the literature on child adjustment in sexual minority parent families (Bos & van Balen, 2008; Golombok et al., 2018), child adjustment problems in the current sample were unrelated to parent-experienced gender minority stress. It is possible that associations were not found due to the heterogeneity of the sample in terms of child age, parental gender identity, and parents’ experiences of gender minority stress (as indicated by the standard deviation value in Table 3), and these factors would benefit from further investigation with larger samples. Homogenous samples, in which variation on multiple sociodemographic factors is restricted, would also be beneficial, however recruitment of homogenous samples of underrepresented groups can be challenging (Bornstein, Jager, & Putnick, 2013). It has also been suggested that the gender minority stress framework may benefit from a broader reconceptualization that acknowledges the institutionalized nature of stressors (Riggs & Treharne, 2017), family support and cultural connectedness (Tan, Treharne, Ellis, Schmidt, & Veale, 2019), and future studies could be cognizant of this.

Increased levels of child adjustment problems were predicted by parental depression, parenting stress, and perceived social support, which is in line with a relational developmental systems approach (Lerner & Callina, 2014; Overton, 2015), which emphasizes the mutually influential relations between individuals and their contexts. Parental depression, parenting stress, and perceived social support are known to be inter-related and associated with child adjustment problems in non-trans families (Dubois-Comtois, Moss, Cyr, & Pascuzzo, 2013; Goodman et al., 2011; McConnell, Breitkreuz, & Savage, 2011; Rodriguez, 2011). That parent–child relationship quality was predicted by parenting stress and parenting quality in the present sample of trans parent families is in line with what would be expected from a relational developmental systems approach, as both constructs are known to be associated with the quality of parent–child relationships (Deater-Deckard, 2004). These findings are also in line with the growing literature on ‘non-traditional’ family forms, which finds that family processes are more influential in determining family functioning than family structure (Golombok, 2015; Lamb, 2012). Scholars working within a relational developmental systems approach have suggested that to gain a more nuanced and holistic understanding of the bidirectional relationships between children and parents, the use of person-centered qualitative approaches may be valuable (Lerner, Johnson, & Buckingham, 2015), and these approaches will likely be important future avenues for research with children in trans parent families (Zadeh et al., 2019).

With regard to parents’ psychological health, just over one-third of trans parents in the current sample had scores indicating depression, with one-quarter having scores that indicated moderate or severe depression. These rates are higher than would be expected in a general population sample (Singleton, Bumpstead, O’Brien, Lee, & Meltzer, 2003) but lower than those found in larger surveys of trans participants (Bockting et al., 2013). Cis parents’ depression scores did not suggest any cause for concern. Most parents scored below the clinical cutoff point for parenting stress and two-thirds of trans parents reported high social support, suggesting that the current sample may be a relatively well-supported one. Higher perceived social support has been highlighted as an important protective factor against depression and anxiety in trans samples (Pflum, Testa, Balsam, Goldblum, & Bongar, 2015) and in a range of cisgender samples across the lifespan (Gariépy, Honkaniemi, & Quesnel-Vallée, 2016). Petit and colleagues have highlighted that for some trans parents the existence of their children may help in times of psychological crisis (Petit, Julien, & Chamberland, 2018), so it is possible that being a parent may act as a protective factor for some trans people.

For trans parents, non-affirmation of gender identity (a type of distal stressor in the gender minority stress framework) was associated with higher parenting stress and lower perceived social support. Although no association was found with depression in the current sample, gender affirmation is known to be associated with well-being (Glynn et al., 2016; Scheim, Perez-Brumer, & Bauer, 2020). Findings of associations between gender affirmation and parenting stress and social support are therefore unsurprising, but nevertheless require replication. These associations were no longer significant once time since communication of gender identity to the child was controlled. Parents who had more recently communicated their gender identity to their child experienced greater non-affirmation and greater parenting stress. As disclosure of a parent’s gender identity involves the negotiation of multiple stresses (Veldorale-Griffin, 2014), these preliminary findings appear to be consistent with the existing literature. It is important to note that because of the cross-sectional nature of the study, it is not possible to draw conclusions about the direction of effects, and future work should incorporate longitudinal designs.

In 20 families (57%), the target child’s parents were no longer together, which is a higher rate than the U.K. national average (Department for Work and Pensions, 2013). However, in only five families was the parents’ separation-related to the trans parent’s gender identity. In 24 (69%) families, the relationship between the child’s parents was rated as amicable, and of those trans parents whose partners knew about their gender identity, 22 (69%) were described as supportive. As such, most of the current sample of children had not experienced high levels of parental animosity toward their trans parent, a factor that may help to explain their good psychological adjustment. In a minority of families, children had experienced high levels of conflict between parents and several parents were not supportive of the trans parent. The effects on family functioning in families in which there are high levels of animosity and unsupportive co-parents require further exploration, as does relationship quality within wider family networks.

A limitation of the study is the sample size, which may be considered small for developmental science research, and in terms of the analyses, limited the number of predictors that could be included in the models. Studies in the future with larger samples would enable more complex models to be tested and a more nuanced understanding to be gained of the factors that contribute to parent–child relationship quality and positive and negative outcomes for children in this particular family type. The small sample size also meant that it was not possible to examine the data with attention to as much within-group diversity as hoped for, either, for instance by analyzing intersectionality quantitatively so as to incorporate and recognize participants’ intersecting identity statuses (Parent, DeBlaere, & Moradi, 2013), or by examining whether differences may exist between sub-groups within the sample. Future studies may wish to explore whether, for example, parental well-being and social support differ between trans parents with different gender identities, or between parents who identify as trans before versus after having children. However, given the hard-to-reach nature of this population, the difficulties involved in recruiting families to take part in research on sensitive topics, around which there may be real or perceived stigma (Nachtigall, Tschann, Szkupinski Quiroga, Pitcher, & Becker, 1997), and the exploratory aims of the study, the sample size may be considered adequate. The sample is larger than other interview-based studies with trans parent families (Chiland et al., 2013; Dierckx et al., 2017; White & Ettner, 2007) and other studies of child adjustment in trans parent families (Church et al., 2014; Freedman et al., 2002; Green, 1978).

A further limitation of the present study is the heterogeneity of the sample, which encompassed a relatively large age range for parents and children (and thus several developmental stages), a diversity of family structures and living arrangements, and parents with a range of gender identities, thus limiting the generalizability of the findings. However, heterogeneous samples are considered acceptable, and even preferable, in exploratory research designs (Henry, 2013). Similarly, trans parent families, and the trans population more broadly, are not homogeneous groups (Brennan et al., 2017), and thus the sample arguably reflects some of this diversity. Furthermore, it has been suggested that there may be higher rates of parenting among trans women (Stotzer et al., 2014), and as the current sample included a high proportion of participants who self-defined as trans women, it could be argued that the sample to some extent reflects the trans parent population. It is also worth noting that, despite the age range of children in the study, spanning 14 years, this is a more focused age-range than several previous studies (e.g., Church et al., 2014; Dierckx et al., 2017; White & Ettner, 2007) in that it does not include adult children.

Although control groups are commonly used in developmental science research, the absence of one in the current study should not necessarily be considered a limitation, first because of its exploratory nature and second because of paradigmatic shifts that are especially relevant to studying this population. Studies examining family functioning in new family forms have historically used a comparative approach, comparing the family type of interest to a majority group defined as a normative ‘control’ (Parent et al., 2013) and have been slow to move beyond this comparative lens (Fish & Russell, 2018). However, this research design has been criticized for its heteronormative bias (van Eeden-moorefield & van Eeden-moorefield, 2018). It has been suggested that researchers could attend more to within-group variation, with within-group designs offering the possibility to contribute to the understanding of queer families in different ways to those offered by between-group designs (Fish & Russell, 2018).

A strength of the study was the multi-method, multi-informant approach that allowed a wider range of measures to be used than has been employed previously with this family type, and for data to be collected from different family members, thus providing a more thorough understanding of the family system. The study is the first to collect quantitative data from school-age children on family functioning in trans parent families. It is well known that children can provide valuable reflections on, and accounts of, their family experiences (Mason & Tipper, 2014), and including both children’s and parents’ perspectives on family relationships in this study goes beyond previous research that had relied on parent or adult-child reports only.

## IMPLICATIONS FOR PRACTICE

As reported in both empirical research (Grant et al., 2011; Pyne et al., 2015), and by several parents in the current sample, there remain cases in the United Kingdom, the United States and Canada of trans parents losing custody or experiencing restrictions of their parental rights on the basis of their gender identity, a situation that appears to have some parallels with that faced by lesbian mothers and gay fathers in the 1970s and 1980s who lost custody of their children on the basis of their sexual orientation (Rivers, 2010). The current findings provide no support for such decisions, showing instead good quality relationships between trans parents and their children, and good psychological adjustment among school-aged children.

The findings from the current exploratory study are of relevance to professionals working with parents and children in trans parent families, as well as to trans parents (and prospective parents) themselves. Findings that children with trans parents showed good quality relationships with their parents and good psychological well-being challenges the practice of restricting parental rights on the basis of a parent’s gender identity. Whereas it had previously been assumed by some professionals that experiencing a parent’s gender transition poses a *“mild to moderate risk”* to children (White & Ettner, 2004), this finding was based on those with experience of clinical samples rather than samples drawn from the general population. Professionals who come into contact with children with trans parents (e.g., counselors, school teachers), should not start with the assumption that living with a trans parent is inherently problematic for children. Given that child adjustment was predicted by parenting stress, parental depression and perceived social support, it may be valuable to focus more on family processes and ensure that psychosocial support is available to those parents who require it.

## ADDRESSES AND AFFILIATIONS

Susan Imrie, Sophie Zadeh and Susan Golombok are at the Centre for Family Research, University of Cambridge.  Kevan Wylie is at the European Federation of Sexology.
